# Mitochondrial Oxidative Stress Impairs Energy Metabolism and Reduces Stress Resistance and Longevity of *C. elegans*

**DOI:** 10.1155/2019/6840540

**Published:** 2019-11-15

**Authors:** Benjamin Dilberger, Stefan Baumanns, Fabian Schmitt, Tommy Schmiedl, Martin Hardt, Uwe Wenzel, Gunter P. Eckert

**Affiliations:** ^1^Institute of Nutritional Sciences, Laboratory for Nutrition in Prevention and Therapy, Biomedical Research Center Seltersberg (BFS), Justus Liebig University Giessen, Schubertstrasse 81, 35392 Giessen, Germany; ^2^Molecular Nutrition Research, Interdisciplinary Research Center, Justus Liebig University Giessen, Heinrich-Buff-Ring 26-32, 35392 Giessen, Germany; ^3^Imaging Unit, Biomedical Research Center Seltersberg (BFS), Justus Liebig University Giessen, Schubertstrasse 81, 35392 Giessen, Germany

## Abstract

**Introduction:**

Mitochondria supply cellular energy and are key regulators of intrinsic cell death and consequently affect longevity. The nematode *Caenorhabditis elegans* is frequently used for lifespan assays. Using paraquat (PQ) as a generator of reactive oxygen species, we here describe its effects on the acceleration of aging and the associated dysfunctions at the level of mitochondria.

**Methods:**

Nematodes were incubated with various concentrations of paraquat in a heat-stress resistance assay (37°C) using nucleic staining. The most effective concentration was validated under physiological conditions, and chemotaxis was assayed. Mitochondrial membrane potential (*ΔΨ*m) was measured using rhodamine 123, and activity of respiratory chain complexes determined using a Clark-type electrode in isolated mitochondria. Energetic metabolites in the form of pyruvate, lactate, and ATP were determined using commercial kits. Mitochondrial integrity and structure was investigated using transmission electron microscopy. Live imaging after staining with fluorescent dyes was used to measure mitochondrial and cytosolic ROS. Expression of longevity- and mitogenesis-related genes were evaluated using qRT-PCR.

**Results:**

PQ (5 mM) significantly increased ROS formation in nematodes and reduced the chemotaxis, the physiological lifespan, and the survival in assays for heat-stress resistance. The number of fragmented mitochondria significantly increased. The ∆*Ψ*m, the activities of complexes I-IV of the mitochondrial respiratory chain, and the levels of pyruvate and lactate were significantly reduced, whereas ATP production was not affected. Transcript levels of genetic marker genes, *atfs-1*, *atp-2*, *skn-1*, and *sir-2.1*, were significantly upregulated after PQ incubation, which implicates a close connection between mitochondrial dysfunction and oxidative stress response. Expression levels of *aak-2* and *daf-16* were unchanged.

**Conclusion:**

Using paraquat as a stressor, we here describe the association of oxidative stress, restricted energy metabolism, and reduced stress resistance and longevity in the nematode *Caenorhabditis elegans* making it a readily accessible *in vivo* model for mitochondrial dysfunction.

## 1. Introduction

Mitochondria supply cellular energy and are key regulators of intrinsic cell death and consequently affect longevity [1, 2]. A link between aging and mitochondrial dysfunction has been well established [2–7]. The “free radical theory” of aging, first proposed by Harman et al., explains aging as a result of the accumulation of cellular damage caused by reactive oxygen species (ROS) [2, 8, 9]. Since mitochondria are the primary source of ROS, Harman himself extended his theory to the “mitochondrial theory of aging” [3, 10]. An imbalance between ROS and cellular stress defence mechanisms accordingly causes a vicious cycle of further mitochondrial dysfunction leading to more ROS, which in turn promotes more damage, an energetic imbalance, and finally triggers cell death and thereby aging. The importance of an equilibrium between ROS and defence mechanism is evidenced by the fact that low concentrations of ROS lead to hormesis with a higher state of stress resistance [11–14].

Investigations on isolated mitochondria in aging nematodes are scarce. Several organisms, ranging from yeast to mice [13, 15–18], have been used to study the effect of alterations within the mitochondrial electron chain (ETC) and longevity [5]. However, *C. elegans* offers distinct advantages compared to other model organisms. Especially its ability as a hermaphrodite to produce identical offspring and its short lifespan make it a powerful tool that has been widely used to investigate longevity-related questions [19, 20]. Since molecular and functional processes associated with mitochondria are highly conserved in species over long evolutionary distances, *C. elegans* represents an outstanding model for aging mechanisms with mitochondria [20]. Specific transcription factors, including *skn-1* (Nrf-2 ortholog), *aak-2* (AMPK ortholog), *atfs-1*, and *sir-2.1* (Sirt1 ortholog), involved in crucial metabolic pathways [21–24], have been identified, connecting alterations in longevity to mitochondrial dysfunction and mitochondrial biogenesis [25, 26].

Compared to *in situ* studies, investigating isolated mitochondria in the context of aging, however, appears to be crucial since it offers clear advantages [27] in an environment free from interfering organelles or reactions [28].

In the present study, paraquat (1,1′-dimethyl-4′4-bipyridinium dichloride; PQ) was used as a well-known stressor of the mitochondrial respiration chain in order to assess the effects of mitochondrial dysfunction on stress resistance and aging. Life span and health span of nematodes, generation of mitochondrial and cytosolic ROS, energy metabolites (ATP, lactate, and pyruvate), and expression of key genes were investigated in whole animals. Mitochondrial integrity and structure as well as activities of ETC complexes and membrane potential (*∆Ψ*m) were evaluated in isolated mitochondria putting the close relation between mitochondrial dysfunction and longevity into a broader context.

## 2. Material and Methods

### 2.1. Chemicals

Chemicals used were of the highest available purity and standard from Sigma-Aldrich (St. Louis, MO, USA) or Merck (Darmstadt, Germany).

### 2.2. Nematode and Bacterial Strain

*C. elegans* wild-type strain N2 was obtained from the Caenorhabditis Genetics Center (University of Minnesota, MN, US). Nematodes were maintained on nematode growth medium (NGM) agar plates seeded with *E. coli* OP50 at 20°C according to standard protocols [29]. For all experiments, synchronous populations were generated through a standard bleaching protocol [30].

### 2.3. Cultivation and Treatment

Synchronous larvae were washed twice in M9 buffer, counted, and adjusted to 10 larvae per 10 *μ*L. Depending on the experiment and on the number needed, nematodes were either raised in 96-well plates (Greiner Bio-One, Frickenhausen, Germany), cell culture flasks (Sarstedt, Nümbrecht, Germany), or OP50 spread NGM plates. For 96-well plates and flasks, OP50-NGM was added as a standardized food source with a volume 4.4-fold of the larvae containing M9 solution. L1 larvae were maintained under shaking at 20°C reaching adulthood within 3 days.

Unless otherwise stated, paraquat was dissolved in M9 and added after reaching young adulthood, 48 h prior to the experiment. M9 was used as control.

### 2.4. Lifespan Assay

To determine the nematode's lifespan at 20°C, a modified protocol from Amrit et al. was applied [31] and synchronized larvae, obtained from egg preparation as stated above, were raised on NGM agar plates spread with standard OP50 *E. coli* culture. After completing the L4 larval stage, 60 healthy animals per group were transferred to fresh NGM *E. coli* containing plates with a sterilized platinum wire. Effectors were incorporated into the OP50 culture with the concentration as needed. Nematodes were transferred to new plates every two days to distinguish between offspring until egg-laying stopped. In line with the separation from eggs and larvae, nematodes were checked for vital signs using a hot platinum wire held next to the animals' heads. Worms showing no reaction to the heat stimulus were considered dead. The lifespan curves were statistically compared using the log-rank test.

### 2.5. Heat-Shock Survival Assay

Approximately 10 nematodes were raised per well in a 96-well microplate as mentioned above. After 48 h of incubation with effectors, time till death was determined using a microplate thermotolerance assay [32]. In brief, nematodes were washed off the wells with M9-buffer into 15 mL tubes followed by three additional washing steps. Each well of a black 384-well low-volume microtiter plate (Greiner Bio-One, Frickenhausen, Germany) was prefilled with 6.5 *μ*L M9 buffer/Tween® 20 (1%*v*/*v*). Subsequently, one nematode was immersed into 1 *μ*L M9 buffer under a stereomicroscope (Breukhoven Microscope Systems, Netherlands). A volume of 7.5 *μ*L SYTOX™ Green (final concentration 1 *μ*M; Life Technologies, Karlsruhe, Germany), which penetrates only into cells with compromised plasma membrane and gets fluorescent after binding to DNA, was added for fluorescent detection. To prevent water evaporation, the plates were sealed with a Rotilabo sealing film (Greiner Bio-One, Frickenhausen, Germany). Heat shock (37°C) was applied and fluorescence measured with a ClarioStar Plate Reader (BMG, Ortenberg, Germany) every 30 min over the course of 17 h. The excitation wavelength was set at 485 nm, and the emission detected at 538 nm.

### 2.6. Chemotaxis Assay

Chemotaxis was assessed using a previously published method [33]. Briefly, agar plates were divided into four quadrants. Sodium acid (0.5 M) was mixed in the same parts with ethanol (95%) as control, or diacetyl (0.5%) as attractant. Either 2 *μ*L of control or attractant solution was added to the center of two opposite quadrants with the same distance to the middle of the plate. Nematodes were washed and separated from larvae as stated above, and a number of approximately 150 animals placed in the plates' center. After 1 h, each quadrant was counted, and a chemotaxis index calculated ((number of attractant – number of control)/number total).

### 2.7. Mitochondrial and Cytosolic ROS Measurement

To determine mitochondrial ROS levels, young adult nematodes were incubated for 48 h with 0.5 *μ*M MitoTracker® Red CM-H_2_XRos (Fisher Scientific, Schwerte, Germany). MitoTracker® Red accumulates to a high extend at the inner mitochondrial membrane showing an increased fluorescence upon elevated ROS mainly associated with mitochondria. To detect cytosolic ROS, nematodes were incubated for 4 h with 25 *μ*M fluorescent probe 2′,7′-dichlorofluorescein diacetate (CM-H_2_DCFDA) (Fisher Scientific, Schwerte, Germany). The probe passively diffuses into cells where it is oxidized by cytosolic reactive oxygen species into its fluorescent form. Paraquat (5 mM) was added 4 h prior to the experiment for both parameters. For epifluorescence microscopy (EVOS FL digital fluorescence microscope, AMG, Bothell, USA), worms were washed with M9-buffer/Tween®20 (1% *v*/*v*) solution and anesthetized by addition of 2 mM levamisole. Nematodes were transferred onto a labelled glass slide and covered with a cover slip. The dye was visualized using the EVOS LED Light Cube RFP, with an excitation at 531 ± 40 nm and an emission at 593 ± 40 nm for MitoTracker® Red and an excitation at 470 ± 22 nm and an emission at 525 ± 50 nm for CM-H_2_DCFDA. Images were taken at a tenfold magnification. For each group, at least 20 nematodes were photographed. The quantification of fluorescence intensity was done using ImageJ (National Institute of Health (NIH)).

### 2.8. Isolation of Mitochondria

To isolate functional mitochondria, nematode populations ranging from 5,000 to 10,000 per group were needed. Nematodes were raised in liquid OP50-NGM culture medium and incubated with effectors as stated above under standardized conditions.

Two-day-old gravid adults were separated from larvae and washed to remove residual bacteria using a self-made separation device with a nylon mesh (Dr. Fill®, Giessen, Germany) before being transferred to ice-cold isolation buffer (300 mM sucrose, 5 mM TES, 200 *μ*M EGTA, pH 7.2) [34].

To obtain a mitochondria-enriched fraction, a Balch Homogenizer (Isobiotec, Heidelberg, Germany) was used [35]. Nematodes were gently passed through the homogenizer chamber with 1 mL glass syringes (SGE Syringe, Trajan, Australia) fitted with a metal luer lock for 5 times. To fracture nematode cuticle, a 12 *μ*m ball clearance was applied. The homogenate was centrifuged at 800 g for 5 minutes at 4°C (Heraeus Fresco 21, Thermo Scientific, Langenselbold, Germany) to sediment debris and larger worm fragments. The mitochondria-containing supernatant was collected and centrifuged at 9,000 g for 10 minutes at 4°C. The crude mitochondria-containing pellet was resuspended in 70 *μ*L swelling buffer (SWB) (0.2 M sucrose, 10 mM MOPS-Tris, 5 mM succinat, 1 mM H_3_PO_4_, 10 *μ*M EGTA, 2 *μ*M rotenone) for measurement of membrane potential (*ΔΨ*m) [34] or 200 *μ*L of mitochondrial respiration medium MirO5 (0.5 mM EGTA, 3 mM MgCl_2_, 60 mM K-lactobionate, 20 mM taurine, 10 mM KH_2_PO_4_, 20 mM HEPES, 110 mM sucrose, 1 g/L BSA, pH 7.1; developed by Oroboros) for high-resolution respiratory experiments [36]. For *ΔΨ*m and respiration measurements, fresh mitochondria were immediately used after preparation. Aliquots were shock frozen in liquid nitrogen for determination of citrate synthase activity and protein content.

### 2.9. Transmission Electron Microscopy

For fixation, 200 *μ*L of fixative (glutaraldehyde (5%) in 0.1 M cacodylate buffer) was added in 200 *μ*L immersed mitochondria, incubated for 30 minutes at room temperature, centrifuged at 9,000 g for 10 minutes, and replaced with fresh fixative (glutaraldehyde (2.5%) in 0.1 M cacodylate buffer). Samples were stored under constant movement overnight at 4°C. Probes were postfixed in 1% OsO_4_ in 0.1 M cacodylate buffer for 45 minutes at room temperature. Before staining with 1% uranyl acetate overnight at 4°C, samples were embedded in low melting temperature gelatine. The gelatine blocks were dehydrated in an ethanol series (10-20 minutes each in 30%, 50%, 70%, 80%, 90%, 96%, 99%, and 99% over molecular sieve) on ice followed by propylene oxide before embedding in Epon and hardened at 60°C for 24 hours. Probes were cut into 80 nm slices, transferred on copper mesh grids and stained with aqueous uranyl acetate followed by lead citrate. Grids were examined with a Leo 912 AB Omega Electron Microscope (Carl Zeiss, Oberkochen, Germany).

To exclude eventual bias, ten pictures for every investigated parameter were taken by a third party and randomized before evaluation. For determination of integrity, mitochondria were divided into four categories (“intact,” outer membrane appears intact and crista structure is visible (a); “mildly fractured,” slight fractures of outer membrane are visible but mitochondria appear overall in a good state (b); “heavily fractured,” outer membrane and crista structure appears damaged (c); “fragmented,” mitochondria are torn in parts or only fragments of former mitochondria are visible (d)). Categorization was conducted by two independent investigators.

### 2.10. Mitochondrial Membrane Potential (*ΔΨ*m)

To determine the mitochondrial membrane potential, a modified protocol of Schmitt et al. was applied [34]. The fluorescent dye rhodamine 123 (Rh123) was used to assess the *ΔΨ*m of 25 *μ*L swelling buffer-resuspended isolated mitochondria in a black 96 well-plate with a ClarioStar Plate Reader (BMG, Ortenberg, Germany). To ensure mitochondrial integrity, the membrane potential was measured for 30 minutes, and after reaching equilibrium, 500 nM FCCP was added to evaluate the *ΔΨ*m-dependent effect on the quenching of Rh123. Results were normalized to protein content.

### 2.11. High-Resolution Respirometry

Respiration experiments were conducted at 20°C using a Clark-type electrode (O2k Oxygraph, Oroboros Instruments, Austria). For each measurement, an aliquot of 80 *μ*L in MirO5-resuspended mitochondria, as described preciously, was inserted into 2 mL of air-saturated MirO5-containing electrode chamber. For analysis, the provided DatLab software (Version 7.0.0.2) was used. To determine mitochondrial function, a complex protocol (developed by Prof. Erich Gnaiger, Oroboros, Innsbruck, Austria) was applied, as previously stated [37].

### 2.12. Citrate-Synthase Activity

Frozen and at -80°C, stored samples were slowly thawed, and a reaction mix containing 0.5 mM oxaloacetate, 0.1 mM 5,5′-dithio-bis-2-nitrobenzoic acid (DTNB), 0.31 mM acetyl coenzyme A, 50 *μ*M EDTA, 5 mM triethanolamine hydrochloride, and 0.1 M Tris-HCl was prepared and pre-heated for 5 minutes at 30°C. To determine citrate synthase (CS) activity, 10 *μ*L of mitochondrial suspension was added, and the resulting complex of DNTB with CoA-SH was measured photospectrometrically at 412 nm [38, 39]. Measurements were performed in triplicate.

### 2.13. Nematode Homogenization

To assess energetic metabolites such as ATP, lactate, and pyruvate, a nematode homogenate was generated. In brief, 4,000 synchronized nematodes were harvested, thoroughly washed, shock frozen, and boiled for 15 minutes prior to sonication to denaturate degrading proteins. After centrifugation at 15,000 g for 10 minutes, supernatants were collected. ATP content was assessed immediately and aliquots stored at -80°C for determination of lactate, pyruvate, and protein content.

### 2.14. ATP Measurement

Intracellular ATP levels were determined using the ATPlite luminescence assay system (Perkin Elmer, Waltham, MA, USA). Luminescence was measured in triplicate following the manufacturer's guidelines with a ClarioStar Plate Reader (BMG, Ortenberg, Germany). Aliquots were stored at -80°C for determination of protein content.

### 2.15. Colorimetric Assessment of Lactate and Pyruvate Content

Frozen homogenate samples were slowly thawed until reaching room temperature. Concentrations of lactate and pyruvate were detected by changes in the NADH content using two colorimetric assay kits from Sigma-Aldrich following the manufacturer's guidelines (Sigma-Aldrich, St. Louis, MO, USA) using a ClarioStar Plate Reader (BMG, Ortenberg, Germany).

### 2.16. Protein Quantification

Protein contents were assessed according to the Pierce™ BCA Protein Assay Kit (Thermo Fisher Scientific, Waltham, MA, USA). Bovine serum albumin was used as a standard.

### 2.17. Quantitative Real-Time PCR

Total RNA was isolated using the RNeasy Mini Kit (Qiagen, Hilden, Germany) according to the manufacturer's guidelines after fracturing the nematodes' cuticle using a Balch Homogenizer with 10 *μ*M clearance. The concentration of RNA was quantified by measuring the absorbance at 260 and 280 nm using a NanoDrop™ 2000c spectrophotometer (Thermo Fisher Scientific, Waltham, MA, USA). RNA purity was assessed with the ratio of absorbance at 260/280 nm and 260/230 nm, respectively. Subsequently, samples were treated with a TURBO DNA-free Kit™ (Thermo Fisher Scientific, Waltham, MA, USA) to remove residual genomic DNA. According to the manufacturer's guidelines, complementary DNA was synthesized from 1 *μ*g total RNA using an iScript cDNA Synthesis Kit (Bio-Rad, Munich, Germany) and temporarily stored at -80°C. qRT-PCR was conducted using a CfX 96 Connect™ system (Bio-Rad, Munich, Germany). Primers were purchased from BioMers (Ulm, Germany). Oligonucleotide primer sequences, primer concentrations, and product sizes are listed in Table 1. All cDNA samples were performed in triplicate after a 1 : 10 dilution with RNase-free water (Quiagen, Hilden, Germany). PCR cycling conditions were an initial denaturation at 95°C for 3 min, followed by 45 cycles of 95°C for 10 s, 58°C for 45 s (with the exception of *aak-2* at 62°C), and extension at 72°C for 29 s. Gene expression levels were analysed by applying the –(2*Δ*ΔC_q_) method using Bio-Rad CfX manager software and normalized to the expression levels of amanitin resistant (*ama-1*) and actin (*act-2*).

### 2.18. Statistics

Values are presented as mean ± standard error of means (SEM). Statistical analyses were performed by applying Student's *t*-test (Prism 8.0 GraphPad Software, San Diego, CA, USA). Statistical significance was defined for *p* values ^∗^*p* < 0.05, ^∗∗^*p* < 0.01, ^∗∗∗^*p* < 0.001, and ^∗∗∗∗^*p* < 0.0001.

## 3. Results

### 3.1. Paraquat Reduces the Survival under Heat-Stress and Lifespan at 20°C

**Figure 1:**
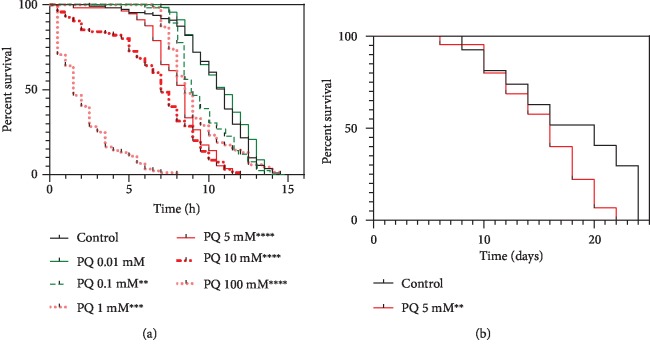
Heat-stress resistance and lifespan of C. elegans are reduced under paraquat exposure. (a) Survival at 37°C was assessed due to the penetration of SYTOX™ Green nucleic acid stain into dead cells. (b) Survival of wild-type C. elegans N2 at 20°C was assessed in the absence and presence of 5 mM paraquat. Log-rank (Mantel-Cox) test; ^∗∗^*p* < 0.01, ^∗∗∗^*p* < 0.001, and ^∗∗∗∗^*p* < 0.0001.

A screening of various paraquat concentrations showed a dose-dependent decline in heat-stress resistance of nematodes (Figure 1(a)). Exposure to 5 mM paraquat, a concentration which caused a significant reduction of stress resistance, also caused a significant reduction in the nematode survival rate at 20°C (Figure 1(b)).

### 3.2. PQ Reduces Chemotaxis

**Figure 2:**
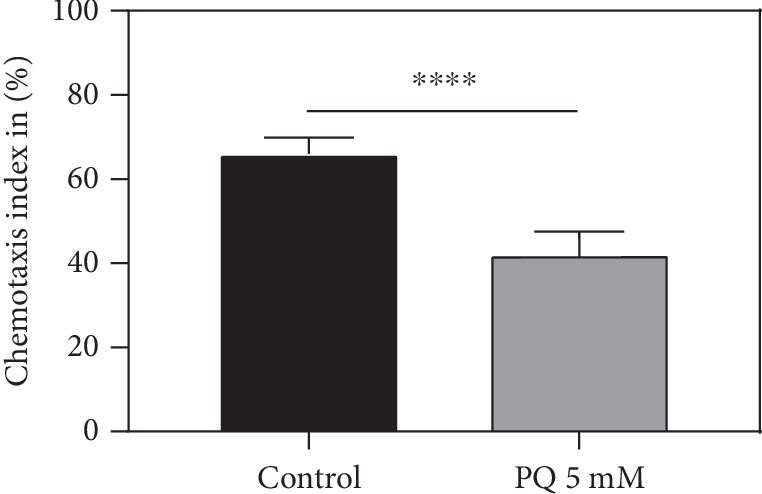
Chemotaxis index after treatment with 5 mM PQ for 48 h. *n* = 5; mean ± SEM; Student's *t*-test; ^∗∗∗∗^*p* < 0.0001.

PQ significantly reduced nematode chemotaxis, resulting in a decreased ability to locate food. Animals insulted with 5 mM PQ appeared 24% less likely to locate the attractant diacetyl compared to control (^∗∗∗∗^*p* < 0.0001) (Figure 2).

### 3.3. Mitochondrial and Cytosolic ROS Measurement

**Figure 3:**
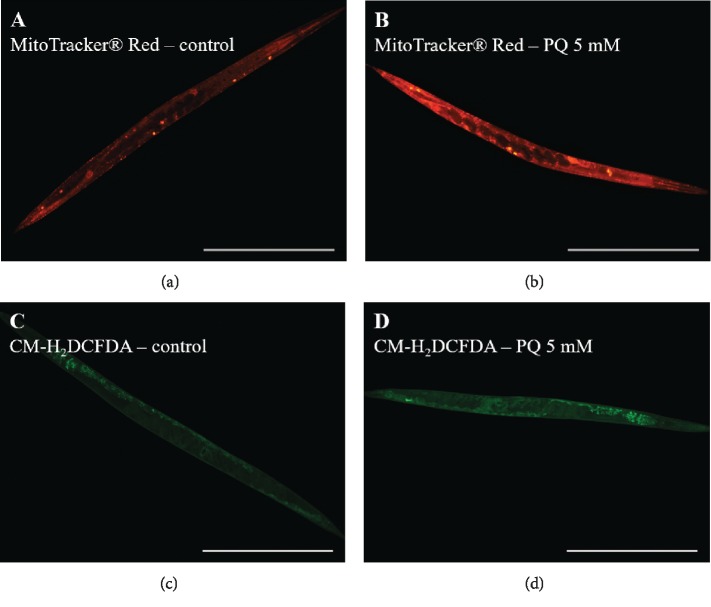
Nematodes, stained with MitoTracker® Red (a, b) for mitochondrial and with CM-H_2_DCFDA (c, d) for cytosolic site of ROS generation, treated for 4 h in the absence (control) or presence of 5 mM PQ. Scaling bar is 400 *μ*M.

To evaluate the effect of PQ on ROS production in the cytosol and mitochondria, especially, of wild-type nematodes, they were stained with two fluorescent markers. MitoTracker® Red was applied to evaluate mitochondrial site of ROS generation and CM-H_2_DCFDA for determination of cytosolic ROS (Figure 3).

**Figure 4:**
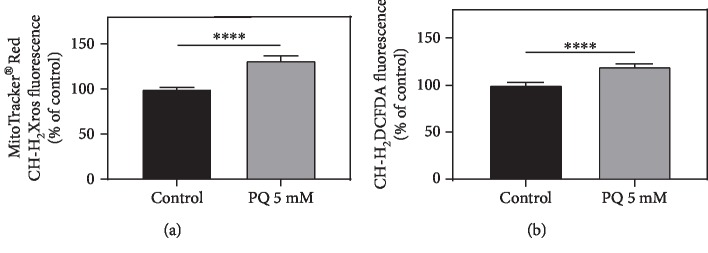
Paraquat (5 mM) increases mitochondrial and cytosolic ROS level in wild-type nematodes after 4 h of exposure. (a) MitoTracker® Red (CM-H_2_Xros) was used to determine mitochondrial and (b) CM-H_2_DCFDA for cytosolic ROS production. Mean ± SEM; Student's *t*-test; ^∗∗∗∗^*p* < 0.0001.

PQ treatment increases mitochondrial ROS level significantly by 31.5% (^∗∗∗∗^*p* < 0.0001) and cytosolic ROS level by 19.4% (^∗∗∗∗^*p* < 0.0001) (Figure 4).

### 3.4. Effects of PQ on the Integrity of Mitochondria

**Figure 5:**
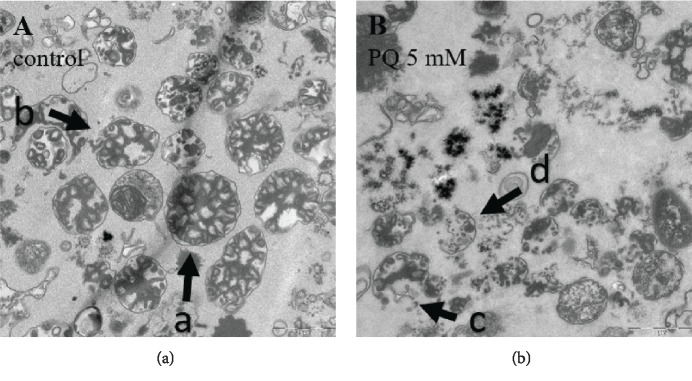
Transmission electron microscopic pictures of isolated mitochondria from C. elegans treated for 48 h in the absence (a; control) or presence of 5 mM PQ (b). Degrees of damage are indicated by exemplary pointers (A = “intact”; B = “mildly fractured”; C = “heavily fractured”; and D = “fractured”).

To elucidate the quality of mitochondria after Balch homogenization as well as the damaging impact of PQ (5 mM) on mitochondrial integrity, we conducted transmission electron mircospy.  Figure 5 shows two representative pictures of mitochondria from control nematodes and those treated with 5 mM. As visualized, PQ causes a significant damage of mitochondria, as evidenced by disrupted membranes and loss of crista structure. Exemplary pointers indicate the different degrees of damage as described in Material and Methods. In brief, “intact” (a), mildly “fractured” (b), “heavily fractured” (c), and “fractured” (d) (Figure 5).

**Figure 6:**
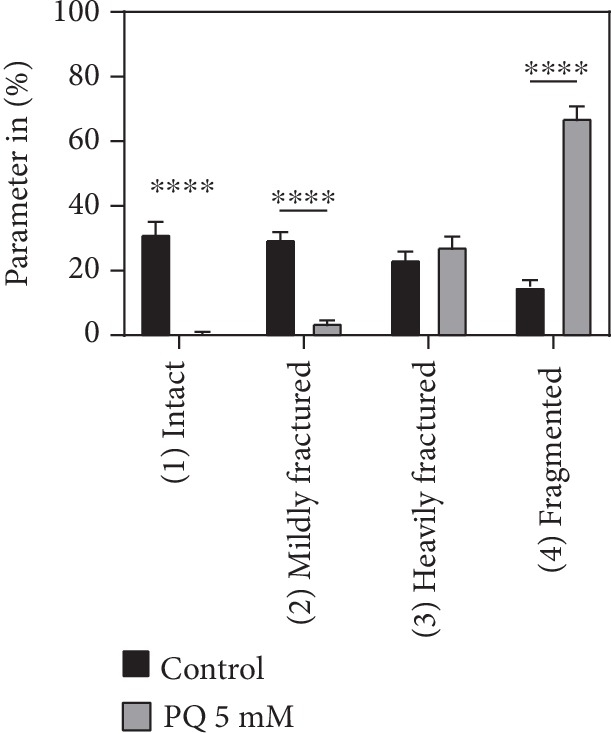
Mitochondria isolated from nematodes after a 48 h incubation in the absence (control) or presence of 5 mM PQ were assessed by transmission electron microscopy and categorized into four categories (intact, mildly fractured, heavily fractured, and fragmented) as described in Material and Methods. Mean ± SEM; Student's *t*-test; ^∗∗∗∗^*p* < 0.0001.

Treatment with PQ resulted in a significantly lower number of fully intact and mildly fractured mitochondria compared to the control. For the heavily fractured category, no significant differences could be observed between the two groups, while the number of fragmented mitochondria significantly increased (Figure 6).

### 3.5. Effect of PQ on Mitochondrial Function

**Figure 7:**
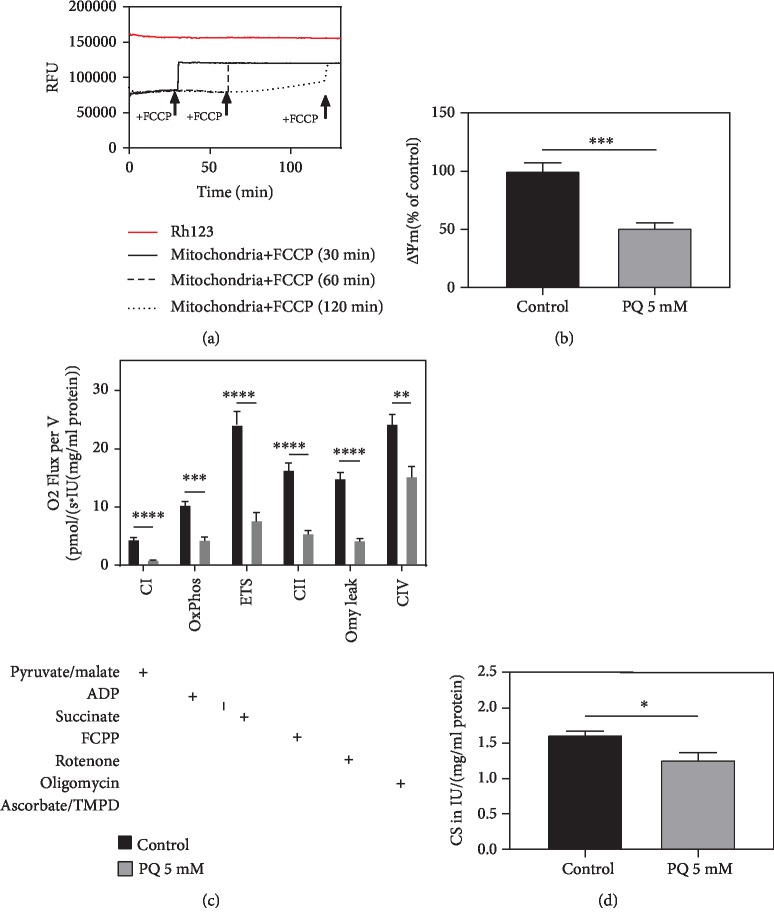
(a) Mitochondrial membrane potential (ΔΨm) over 130 minutes assessed by fluorescent dye rhodamine 123 (Rh123). Short circuit of ΔΨm was achieved by the addition of FCCP after 30, 60, and 120 minutes. (b) ΔΨm (MMP), assessed by mitochondria-dependent increase of Rh123 fluorescence in percentage, after PQ treatment (5 mM) for 48 h, detected with a ClarioStar Plate Reader (BMG, Ortenberg, Germany). (c) Respiration of isolated mitochondria from C. elegans normalized to (d) citrate synthase activity in international units IU/(mg/mL protein). Activity of respiration complexes was measured using an O2k Oxygraph (Oroboros, Innsbruck, Austria). Addition of substances into the Oxygraph's chambers is indicated with a plus sign (+). Mean ± SEM; Student's *t*-test; ^∗^*p* < 0.05, ^∗∗^*p* < 0.01, ^∗∗∗^*p* < 0.001, and ^∗∗∗∗^*p* < 0.0001.

To ensure mitochondrial integrity, its membrane potential (*ΔΨ*m) was measured over the course of 130 minutes after isolation with fluorescent dye rhodamine 123 (Rh123). Addition of 500 nM carbonyl cyanide-4-(trifluoromethoxy)phenylhydrazone (FCCP), at three different time points to aliquots of the same isolation, was used to depolarize the inner mitochondrial membrane and to abolish *ΔΨ*m, after 30, 60, and 120 minutes [34].  Figure 7(a) shows a maximum fluorescence of Rh123 alone and a constant signal after quenching of the fluorescent dye in intact mitochondria beyond the time point of 60 minutes and slowly increasing afterwards. Addition of FCCP depolarizes *ΔΨ*m resulting in an increased Rh123 signal after all time points to the same level.

PQ (5 mM) significantly decreased ΔΨm by 49% compared to the control (Figure 7(b)). PQ impaired the mitochondrial respiratory chain and significantly reduced the activity of CI, CII, and CIV (Figure 7(c)).

The ratio between ETS and leak respiration after oligomycin addition, known as respiratory control ratio (RCR), is an accepted indicator of an increased proton gradient for ATP synthesis via complex V [37]. RCR was not altered after PQ incubation (data not shown).

The activity of citrate synthase (CS), as part of the Krebs cycle, is an established mitochondrial matrix marker [40]. PQ significantly reduced CS activity (Figure 7(d)) indicating a reduced mitochondrial mass [41].

### 3.6. PQ Decreases the Levels of Pyruvate and Lactate

**Figure 8:**
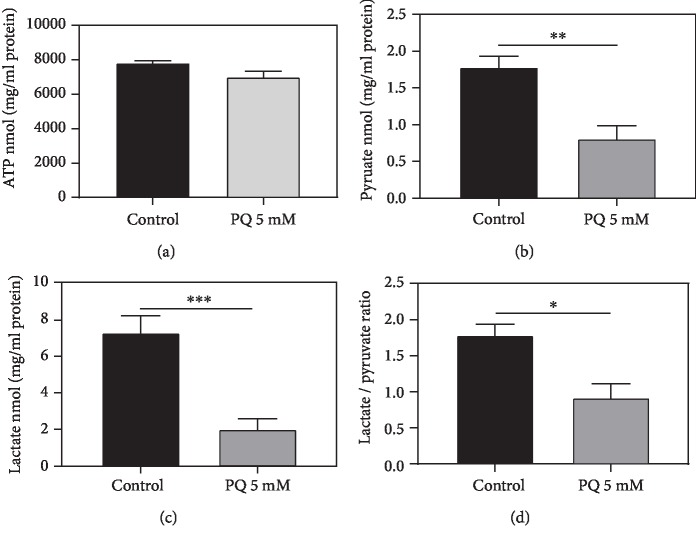
Determination of intracellular ATP levels (a), pyruvate (b), lactate (c), and lactate/pyruvate ratio (d) of wild-type C. elegans exposed to PQ (5 mM) or not (control). ATP levels were assessed using an ATPlite luminescence assay, and lactate and pyruvate using two colorimetric assay kits. Values were normalized to protein concentrations. *n* = 8; Mean ± SEM; Student's *t*-test; ^∗^*p* < 0.05, ^∗∗^*p* < 0.01, and ^∗∗∗^*p* < 0.001.

Next, effects on energy metabolites were determined. Although PQ significantly impaired *ΔΨ*m, respiratory chain, and mitochondrial integrity, ATP levels were not significantly reduced (Figure 8(a)). The energetic metabolites pyruvate and lactate were significantly decreased (Figures 8(b) and 8(c)). The decline of lactate was stronger than the reduction of pyruvate, resulting in a significantly decreased lactate/pyruvate ratio after PQ treatment (Figure 8(d)), indicating a lower glycolytic turnover [42].

### 3.7. PQ Causes an Upregulation of Genes Relevant for Longevity and Mitochondrial Biogenesis

**Figure 9:**
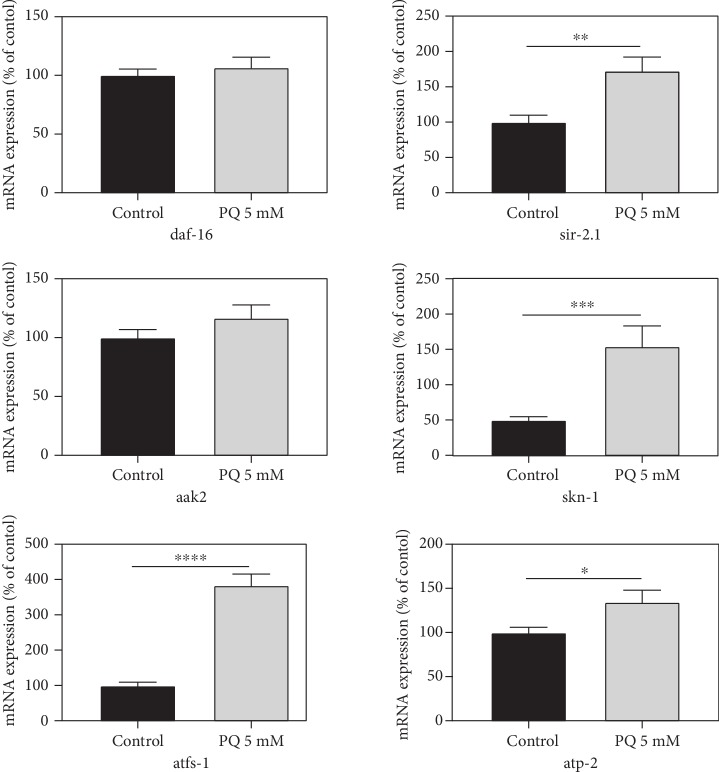
Relative normalized mRNA expression levels of longevity- and mitochondrial biogenesis-related genes daf-16, sir-2.1, aak-2, skn-1, atfs-1, and atp-2 in C. elegans in the absence or presence of PQ (5 mM); Mean ± SEM; Student's *t*-test; ^∗^*p* < 0.05, ^∗∗^*p* < 0.01, ^∗∗∗^*p* < 0.001, and ^∗∗∗∗^*p* < 0.0001; Results are normalized to the mRNA expression levels of amanitin resistant (ama-1) and actin (act-2).

PQ affected the expression levels of several longevity- and mitochondrial biogenesis-related genes in *C. elegans*. The ortholog of human forkhead box O1 *daf-16* known to be relevant for stress resistance, was insignificantly increased by 7%, likewise to *aak-2* (AMP-activated kinase) which was increased by 17% at its transcript level. *Sir-2.1*, which also plays a prominent role for longevity and stress-resistance and encodes an ortholog of human Sirtuin 1, was significantly increased by 73%. Marker genes for mitochondrial biogenesis, i.e., *skn-1*, *atfs-1*, and *atp-2*, were all significantly upregulated at the mRNA level by paraquat (Figure 9).

## 4. Discussion

*C. elegans* represents a well-established model especially for investigations of longevity and genetic variations [43–46]. Mitochondria have been unravelled as organelles with a great impact on longevity and stress resistance [47]. While mitochondrial investigations in nematodes are conducted predominantly using fluorescent staining methods [48–50], mitochondrial isolation protocols are scarce [43]. In this study, we not only present data supporting the connection between mitochondrial function and longevity but also an easy and suitable way to isolate functional mitochondria. Questions concerned with the organelles' function are more distinct in a cell-free environment, free from intervening influences [28].

Paraquat (PQ) was used to induce oxidative stress resulting in mitochondrial dysfunction to display significant effects on the nematodes' lifespan and their respiratory chain capacity. PQ represents a known stress inducer impairing all respiratory chain complexes but most prominently complex I. In a reduced state, it uses oxygen as an oxidant producing the radical superoxide, mediating PQ toxicity [51]. PQ has been commonly used to trigger mitochondrial stress and dysfunction in *C. elegans* [52], as well as to reduce lifespan [53], and was therefore preferred over other known inducers of oxidative stress, as for example juglone, creating ROS through an increased apoptosis [54], or rotenone which specifically blocks complex I and therefore is commonly used for Parkinson's disease models [55].

Our goal was to identify links and causalities between mitochondrial dysfunction and a reduced lifespan and healthspan, as well as to establish a platform for future investigations concerned with potentially beneficial effects of nutrients or pharmaceuticals. We used a Balch Homogenizer and confirmed that the use of this technique represents a highly efficient and powerful tool to isolate mitochondrial fractions with a high quality [34].

### 4.1. Linking Longevity and Mitochondrial Function

In different experimental settings, various concentrations of PQ ranging from 0.2 to 25 mM were used [56–59]. Thus, identifying an optimal concentration for our investigations was the initial step. In a heat-stress resistance assay, we tested PQ concentrations ranging from 0.01 mM to 100 mM to establish their effects on the nematodes' survival at 37°C. A concentration-dependent decline in the nematodes' ability to tolerate heat stress of 37°C could be observed. In our experiments, a concentration of 5 mM PQ was the lowest to reach a level of significance of ^∗∗∗∗^*p* < 0.0001, lowering the median survival from 11 h to 8.5 h and was therefore selected for the following investigations. Interestingly, even though not significant, PQ at a concentration of 0.01 mM led to a slightly increased tolerance of heat stress in wild-type nematodes. To validate PQ 5 mM, as a concentration high enough to alter the nematodes' lifespan, supposedly through mitochondrial stress, we tested this concentration in a survival experiment under physiological conditions. Again, PQ significantly decreased the nematodes' survival by 20% (^∗∗^*p* = 0.0026) compared to control.

Similar to our results, Wu et al. used PQ as an oxidative stressor in an thermotolerance assay, resulting in a reduced survival time of nematodes [59]. While PQ is often used to generate oxidative stress [60], to our best knowledge, the effect of sole PQ exposure on nematode survival under physiological conditions has only been investigated with a concentration of 0.25 mM [58]. This low-dose exposure resulted in an extended lifespan, comparable to our heat-stress survival assay, showing a slightly increased stress resistance after 0.01 mM PQ exposure, suggesting a mitohormetic effect [61, 62]. Generally, a close relation between elevated reactive oxygen species and shortened lifespan expectancy can be drawn throughout investigations and species [63, 64], which is in line with our findings.

Nematodes retrive their food through chemotaxis [33, 65]. Mitochondrial dysfunction not only effects longevity as such but also impairs neuronal function and consequently the animals' sensory ability of tracking food. This closely links chemotaxis and longevity through cellular and neuronal impairment caused by oxidative stress [66]. PQ significantly decreased chemotaxis in *C. elegans*, thus further strengthening the connection between mitochondrial dysfunction and longevity from another point of view. To our best knowledge, the effect of PQ on chemotaxis has never been invested but fits our previous findings and is coherent with the results of Wu et al. showing chemotaxis deficits caused through A*β*-mediated oxidative stress [67].

### 4.2. Isolation and Qualification of Isolated Nematode Mitochondria

Mitochondrial investigations in *C. elegans* so far have mostly been restricted to measuring overall oxygen consumption, using, for example, an O2k Oxygraph from Oroboros, or fluorescent staining of whole cells or nematodes targeting ROS generation or membrane potentials [68–71]. Studies focusing on isolated mitochondria, however, have been scarce [43, 71]. Isolated mitochondria have distinct advantages compared to whole cell or organism systems, since they allow investigations free from interfering organelles or cellular reactions and make it possible to draw direct causalities [27, 28]. A challenge is the nematodes' resilient cuticle making it hard to gather sufficient and especially efficient mitochondria. Several techniques have been described, ranging from grinding worms with a pestle after snap freezing in liquid nitrogen, “bead beating” with glass beads, to mechanically disrupting the nematodes' cuticle, or sonication. Nonetheless, each method suffers from one or more limitations. Grinding requires relatively large sample sizes, which are easily lost [72]. When applying “bead beating” or sonication, samples are prone to denaturation as samples heat up during the process and potentially damaging mitochondria [73]. Furthermore, sonication is unable to produce intact nucleic acids.

Balch homogenization is able to effectively break *C. elegans* cuticles of any stages [72]. The system is able to produce functional proteins, qRT-PCR quality mRNA, and intact mitochondria of a high quality and intactness. Through changeable ball clearance sizes, or number of syringe passes, homogenization roughness can be controlled [34, 74].

Mitochondria isolated with a 12 *μ*m ball clearance appear more intact, after electron microscopy, compared to isolates gathered with a ball clearance leaving only a 6 *μ*m gap (see Supp. [Supplementary-material supplementary-material-1]). A semiquantitative and double-blinded counting validates the first impression and shows that especially percentage numbers in the “heavily fractured” (32%) and “fragmented” (25%) categories are higher for mitochondria isolated with a 6 *μ*m ball clearance, compared to 12 *μ*m. Here, only 24% was “heavily fractured” and 15% fragmented. Rougher homogenization using a 6 *μ*m clearance results in only 15% fully intact mitochondria compared to 32% for a 12 *μ*m clearance (see Supp. [Supplementary-material supplementary-material-1]).

The applicability of isolation of mitochondria with a 12 *μ*m clearance was further evidenced by addition of cytochrome *c* which would enter, if fractured, the outer mitochondrial membrane resulting in an increased respiration rate [75]. No changes of respiration, however, could be observed after isolation with a 12 *μ*m clearance, supporting the integrity of the outer membrane, whereas a significant increase in respirational flux was measured after isolation with a 6 *μ*m clearance (see Supp. [Supplementary-material supplementary-material-1]).

Since none of our described experimental procedures takes more than 30 minutes after isolation, mitochondrial stability over that time period needed to be ensured. We could demonstrate a stable *ΔΨ*m over more than 60 minutes giving more than twice the time needed for all the experiments described. Furthermore, this verifies the 12 *μ*m ball clearance and 5 strokes applied, as valid parameters to generate a sufficient and intact mitochondrial fraction, as evidenced by transmission electron microscopy. Treatment of nematodes with 5 mM PQ, however, caused a significant increase in heavily fractured and fragmented mitochondria characterized by membrane rupture and damaged crista structure.

### 4.3. Impact of Paraquat on Mitochondrial Function

Paraquat at a concentration of 5 mM was identified to reduce significantly the stress resistance as well as longevity in wild-type nematodes. This was associated with a significantly reduced mitochondrial membrane potential (*ΔΨ*m) after 48 h. A similar decrease in *ΔΨ*m, after PQ treatment, was observed by Zhang et al. and Wu et al., while the latter also described a decreased expression of complex I, II, and III genes of the mitochondrial respiratory chain (ETC) [76, 77]. The latter findings are in line with the results of our respiration experiments, where PQ significantly decreased respiration rates of complex I and II. A decreased activity could be observed in our experiments also for the respiratory complexes CI and CII as well as for the OxPhos, ETS, and OmyLeak. Interestingly, complex IV was not altered in its activity. Citrate synthase activity (CS) represents an established indicator for the mitochondrial mass [40]. Thus, the decreased CS activity in our experiments indicates that PQ reduced the mitochondrial mass. These results fit to reports describing a reduced respiratory capacity and a reduced mitochondrial mass after 4 mM PQ exposure in a PC12 cell model [78] or decreased respiration rates in zebra fish [79].

To validate an increased ROS generation by PQ as the reason for mitochondrial dysfunction, we investigated mitochondrial and also cytosolic ROS levels. Since PQ treatment for 48 h decreased intestinal pumping of nematodes causing an insufficient uptake of both fluorescent markers (data not shown), we had to decrease the time of exposure to 4 h. Nevertheless, also after this short incubation period, ROS levels at both locations were significantly increased which is in line with previous findings [14, 58, 59, 80, 81].

According to the observed dysfunction of mitochondria, it appeared reasonable to suggest that PQ causes limitations in the generation of ATP and by the accumulation of substrates also reduces the glycolytic flux. Interestingly, only slightly decreased ATP concentrations were found. This might be explained by the increased ATP synthase activity as a consequence of enhanced *atp-2* mRNA levels observed here. Significant ATP depletion of approximately 20% was found, however, at higher concentrations of 10 mM PQ in nematodes [76]. Pyruvate and lactate levels, however, were significantly decreased after PQ insult in our studies. Similar results have already been shown for glycolytic metabolites after PQ exposure in SK-N-SH cells [82].

To elaborate genetic pathways affected by PQ exposure, we assessed the mRNA levels of *daf-16*, *aak-2*, and *sir-2.1*, three genes with relevance for longevity and stress-resistance longevity [70, 77, 78]. While *daf-16* and *aak-2* were only slightly elevated due to PQ, expression of the human Sirtuin 1 ortholog *sir-2.1* was significantly increased by 73%. *Daf-16* as key modulator of longevity in *C. elegans*, is activated, amongst others, by *sir-2.1* and *aak-2* [83]. The lack of enhanced expression of *daf-16* lets us suggest that the enhanced expression of *sir-2.1* is not sufficient to activate *daf-16* expression in the presence of PQ. However, another gene important for stress resistance and also for mitogenesis, which was found to be significantly upregulated, is the *nrf-2* homolog *skn-1. Skn-1* upregulation was also found to increase the activity of PGC1*α*, the mammalian key regulator of mitogenesis, which, however, has no direct homolog in the nematode [50, 84]. Adaptations to compensate for the PQ-induced stress are evident finally also by enhanced expression of *atfs-1*, a key regulator of mitochondrial unfolded protein response (UPR^mt^).

## 5. Conclusions

Using paraquat as a stressor, we here describe the close association of oxidative stress, restricted energy metabolism, reduced stress resistance, and longevity in the nematode *Caenorhabditis elegans*, focusing on isolated mitochondria, making it a readily accessible *in vivo* model for mitochondrial dysfunction.

## Figures and Tables

**Table 1:** Oligonucleotide primer sequences and product sizes for quantitative real-time PCR. Concentration was 0.1 *μ*M for all primers.

Primer	Sequence	Product size (bp)
*Aak-2*	5′-tgcttcaccatatgctctgc-3′ 5′-gtggatcatctcccagcaat-3′	219

*Ama-1*	5′-ccaggaacttcggctcagta-3′ 5′-tgtatgatggtgaagctggcg-3′	85

*Act-2*	5′-cccactcaatccaaaggcta-3′ 5′-gggactgtgtgggraacacc-3′	168

*Atfs-1*	5′-tcggcgatcgatcagctaac-3′ 5′-agaatcagttcttggattagggga-3′	75

*Atp-2*	5′-tccaagtcgctgaggtgttc-3′ 5′-aggtggtcgagttctcctga-3′	151

*Daf-16*	5′-tcctcattcactcccgattc-3′ 5′-ccggtgtattcatgaacgtg-3′	175

*Sir-2.1*	5′-tggctgacgattcgatggat-3′ 5′-atgagcagaaatcgcgacac-3′	179

*Skn-1*	5′-acagggtggaaaaagcaagg-3′ 5′-caggccaaacgccaatgac-3′	246

bp: base pairs; Conc: concentration.

## Data Availability

The dataset generated during this study is available from the corresponding author on reasonable request.

## Supplementary Materials

Supplementary Materials Supp.1: transmission electron microscopic pictures of mitochondria from C. elegans isolated with a 12 *μ*m (A) or 6 *μ*m ball clearance (B). Degrees of damage are indicated by exemplary pointers (a = “intact”; b = “mildly fractured”; c = “heavily fractured”; and d = “fractured”). Supp. 2: mitochondria isolated from nematodes with 12 *μ*m or 6 *μ*m clearance were assessed by transmission electron microscopy and categorized into four categories (intact, mildly fractured, heavily fractured, and fragmented) as described in Material and Methods. Isolation with 6 *μ*m ball clearance resulted in significantly less intact as well as more fragmented mitochondria. Mean ± SEM; Student's *t*-test; ^∗^*p* < 0.05, ^∗∗^*p* < 0.01. Supp. 3: cytochrome c addition, as a marker for intactness of the outer mitochondrial membrane, increases respiration after isolation with a 6 *μ*m compared to a 12 *μ*m clearance, indicating a fractured outer membrane. *n* = 5; Mean ± SEM; Students *t*-test; ^∗∗∗^*p* < 0.001.
