# Supplementary material for: Mitochondrial Oxidative Stress Impairs Energy Metabolism and Reduces Stress Resistance and Longevity of C. elegans
Source: Oxid Med Cell Longev. 2019 Nov 15;2019:6840540. doi: 10.1155/2019/6840540 (PMC6885289; doi:10.1155/2019/6840540)
Supplement: Supplementary Materials — Supp.1: transmission electron microscopic pictures of mitochondria from C. elegans isolated with a 12 μm (A) or 6 μm ball clearance (B). Degrees of damage are indicated by exemplary pointers (a = “intact”; b = “mildly fractured”; c = “heavily fractured”; and d = “fractured”). Supp. 2: mitochondria isolated from nematodes with 12 μm or 6 μm clearance were assessed by transmission electron microscopy and categorized into four categories (intact, mildly fractured, heavily fractured, and fragmented) as described in Material and Methods. Isolation with 6 μm ball clearance resulted in significantly less intact as well as more fragmented mitochondria. Mean ± SEM; Student's t-test; ∗p < 0.05, ∗∗p < 0.01. Supp. 3: cytochrome c addition, as a marker for intactness of the outer mitochondrial membrane, increases respiration after isolation with a 6 μm compared to a 12 μm clearance, indicating a fractured outer membrane. n = 5; Mean ± SEM; Students t-test; ∗∗∗p < 0.001. [file 6840540.f1.pdf]

Supplementary Material:

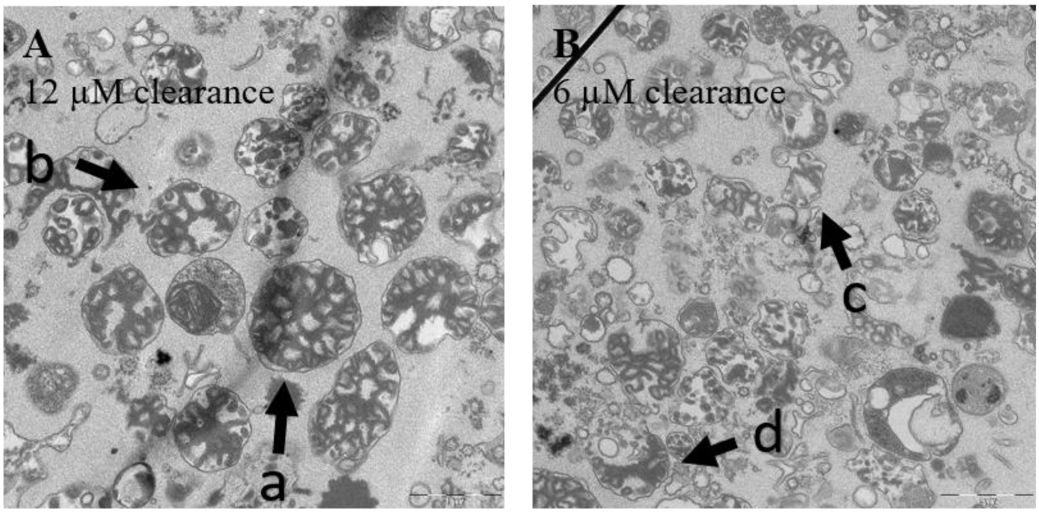

Supp.1: *Transmission electron microscopic pictures of mitochondria from C. elegans isolated with a 12µm (**A**) or 6µm ball clearance (**B**). Degrees of damage are indicated by exemplary pointers (**a** = "intact"; **b** = "mildly fractured"; **c** = "heavily fractured" and **d** = "fractured").*

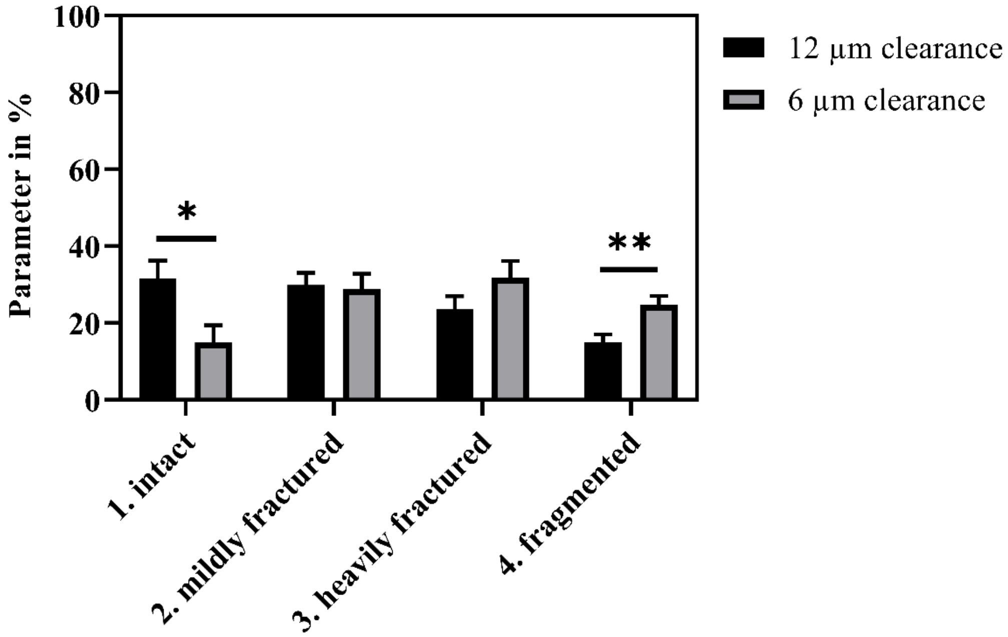

***Supp. 2**: Mitochondria isolated from nematodes with 12 µm or 6 µm clearance were assessed by transmission electron microscopy and categorized into four categories (intact, mildly fractured, heavily fractured and fragmented) as described in the Methods section. Mean ± SEM; students t-test; p* < 0.05, p** < 0.01.*

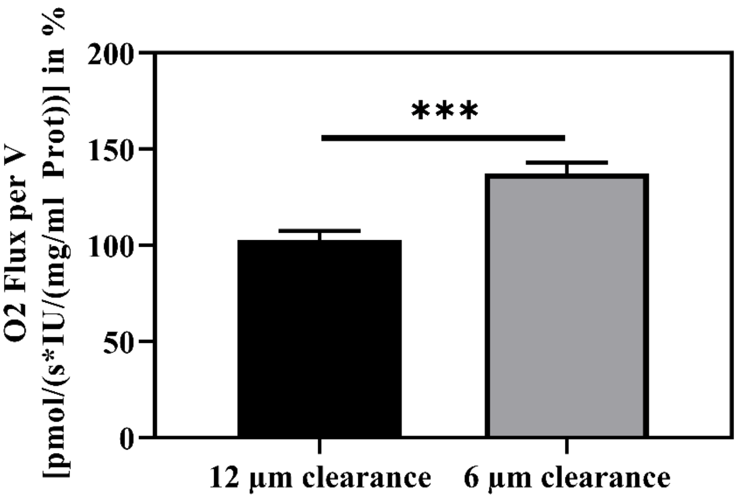

Supp. 3**: Cytochrome c addition, as a marker for intactness of the outer mitochondrial membrane, increases respiration after isolation with a 6 µm compared to a 12 µm clearance, indicating a fractured outer membrane. n = 5; Mean ± SEM; students t-test; p < 0.001.*
